# TAFRO syndrome as a cause of glomerular microangiopathy: a case report and literature review

**DOI:** 10.1186/s12882-019-1574-9

**Published:** 2019-10-17

**Authors:** Yoshikuni Nagayama, Mizuki Yamano, Motoka Yagame, Tomoyuki Nariyama, Mikiko Takahashi, Masashi Kawamoto, Katsuyuki Matsui

**Affiliations:** 1 Department of Internal Medicine IV, Teikyo University School of medicine, University Hospital, Mizonokuchi, Kawasaki, Japan; 2 Department of Diagnostic Pathology, Teikyo University School of medicine, University Hospital, Mizonokuchi, Kawasaki, Japan

**Keywords:** Castleman disease, Interleukin (IL)-6, Membranoproliferative glomerulonephritis (MPGN), TAFRO syndrome, Thrombotic microangiopathy (TMA), Tocilizumab, Vascular endothelial growth factor (VEGF)

## Abstract

**Background:**

TAFRO syndrome is a systemic inflammatory disorder that manifests as thrombocytopenia (T), anasarca (A), fever (F), reticulin fibrosis (R), and organomegaly (O). Renal dysfunction is frequently complicated with TAFRO syndrome, however, it is challenging to perform kidney biopsy in patients with TAFRO syndrome in the presence of thrombocytopenia. Renal histology in TAFRO syndrome mainly shows membranoproliferative glomerulonephritis (MPGN)-like lesions or thrombotic microangiopathy (TMA)-like glomerulopathy. We review our case and previous reports of TAFRO syndrome with kidney biopsy findings and discuss the renal pathophysiology of TAFRO syndrome.

**Case presentation:**

We describe a previously healthy 48- year-old woman with TAFRO syndrome. Kidney biopsy performed before the treatment showed diffuse global endocapillary proliferative changes with endothelial cell swelling, double contours of partial capillary walls, and mesangiolysis, consistent with TMA-like glomerulopathy. Glucocorticoid therapy including steroid pulse was ineffective and she developed anasarca, renal dysfunction and oliguria. Hemodialysis was required. However, the anti-Interleukin (IL)-6 receptor antibody (tocilizumab) therapy was very effective. An increase in urinary volume was achieved about 2 weeks after the tocilizumab therapy and hemodialysis was discontinued. To investigate the renal pathophysiology of TAFRO syndrome, we performed immunohistological staining of vascular endothelial growth factor (VEGF)-A, CD34, and D2–40, in our case and a normal control kidney. Glomerular VEGF-A was especially positive in podocytes both, in the control and in the case, with no significant difference and there was a significant increase of VEGF-A staining area in the cortical peritubular capillaries in the case. Both glomerular and renal cortical CD34 expression were significantly decreased in our case. D2–40 expression in cortex was not significantly different.

**Conclusions:**

We reviewed our case and other 10 previous reports about renal biopsy findings in TAFRO syndrome and found that glomerular microangiopathy was a common finding. IL-6-VEGF-axis-induced glomerular microangiopathy may play a crucial role in developing acute kidney injury in TAFRO syndrome and the anti-IL-6 receptor antibody therapy may be useful for TAFRO syndrome refractory to glucocorticoids. About the pathophysiology of VEGF in TAFRO syndrome, VEGF balance in the glomerulus and perhaps in the peritubular capillary system as well may be critical. Further investigation is needed.

## Background

TAFRO syndrome is a systemic inflammatory disorder that manifests as thrombocytopenia (T), anasarca (A), fever (F), reticulin fibrosis (R), and organomegaly (O) [1]. In 2015, the Japanese diagnostic criteria and treatment strategy of TAFRO syndrome were published [2]. A diagnosis of TAFRO syndrome requires the fulfillment of all three major criteria and at least two of the four minor criteria as follows: The major criteria include (1) Anasarca, including pleural effusion, ascites, and general edema, (2) Thrombocytopenia defined as pre-treatment platelet count ≦100 × 10^3^/μL, and (3) Systemic inflammation defined as fever of unknown etiology (body temperature > 37.5 °C and/or serum C-reactive protein (CRP) concentration ≧2 mg/dL, The minor criteria include (1) Castleman disease-like features on lymph node biopsy, (2) Reticulin myelofibrosis and/or increased number of megakaryocytes in the bone marrow, (3) Mild organomegaly, including hepatomegaly, splenomegaly and lymphadenopathy, and (4) Progressive renal insufficiency. Malignancies; autoimmune disorders; infectious disorders; polyneuropathy, organomegaly, endocrinopathy, M-protein, skin change -syndrome; immunoglobulin G4 (IgG4)-related disease; hepatic cirrhosis; and thrombotic thrombocytopenic purpura (TTP) /hemolytic uremic syndrome must be excluded. Recently, TAFRO syndrome was proposed as a distinct subtype of human herpes virus (HHV)-8-negative multicentric Castleman disease [3]. Renal dysfunction is frequently complicated with TAFRO syndrome. In the presence of thrombocytopenia, it is challenging to perform kidney biopsy in patients with TAFRO syndrome. However, kidney biopsy is being performed for an increasing number of TAFRO syndrome patients. Renal histology in TAFRO syndrome mainly shows membranoproliferative glomerulonephritis (MPGN)-like lesions or thrombotic microangiopathy (TMA)-like glomerulopathy [4–13]. We report a case of TAFRO syndrome with TMA-like glomerulopathy that was successfully treated with an anti-interleukin (IL)-6 receptor antibody (tocilizumab) and discuss the renal pathophysiology of TAFRO syndrome.

## Case presentation

### Clinical history and initial laboratory data

A previously healthy 48-year-old woman underwent a medical check at our hospital for whole body edema (face, extremities, and abdomen). A few weeks previously, she had experienced headache and fever. Laboratory findings showed proteinuria (1.57 g/g・creatinine using spot urine), renal dysfunction (serum creatinine 1.08 mg/dL), and higher CRP level (16.7 mg/dL). A plain computed tomography revealed multiple lymphadenopathy. She was admitted to our hospital for further investigation. Her clinical findings on admission were as follows: blood pressure, 148/92 mmHg; pulse rate, 100/min; body temperature, 39.3 °C; height, 156 cm; and weight, 74 kg. She was alert and had mild facial, upper and lower-extremity, and abdominal edema. She reported slight upper-abdominal pain. She had no superficial lymphadenopathy, joint pain, neurological findings, or skin lesions, and an examination of her heart and lungs was unremarkable. Laboratory findings on admission are shown in Table 1. Urinalysis showed proteinuria (protein 3+ and blood ± by dipstick, and some ovary fatty bodies), and the blood tests revealed hyperleukocytosis (77.6% neutrophils, 11.1% lymphocytes, 10.8% monocytes), abnormal coagulation, hypoproteinemia, renal dysfunction [serum creatinine, 1.32 mg/dl; estimated glomerular filtration rate (eGFR), 34.84 ml/min/1.73 m^2^], elevated alkaline phosphatase, elevated γ-glutamyl transpeptidase, and elevated CRP. eGFR was calculated by using the Japanese eGFR equation (eGFR = 194 × serum creatinine ^− 1.094^ × Age ^− 0.287^ × 0.739) [14]. The patient did not have hypergammaglobulinemia or hypocomplementemia, and soluble IL-2 receptor was elevated. The patient showed negative for various autoantibodies, hepatitis B virus, hepatitis C virus, human immunodeficiency virus (HIV), HHV-8, and tuberculosis infections. IL-6 and vascular endothelial growth factor (VEGF) were remarkably elevated. ADAMTS13 (a disintegrin-like and metalloproteinase with thrombospondin type 1 motifs 13) activity was decreased to 0.633 IU/mL, which was not low enough to be shown in typical TTP.

**Table 1:** Laboratory findings on admission

Parameter	Value (reference range)	Parameter	Value (reference range)
Hematology		Urine BJP	negative
WBC count, /μL	10,160 (3500–9100)	Immunology	
Hemoglobin, g/dL	12.6 (11.3–15.2)	IgG, mg/dL	842 (820–1740)
Platelet count, 10^4^/μL	20.5 (13.0–36.9)	IgG4, mg/dL	28 (5–117)
PT, %	65.5 (80–100)	IgA, mg/dL	166 (90–400)
APTT, seconds	40.7 (24–39)	IgM, mg/dL	100 (52–270)
FDP, μg/mL	26 (< 5)	IgE, IU/mL	23 (< 170)
Blood chemistry		C3, mg/mL	1.62 (0.86–1.6)
Cr, mg/dL	1.32 (0.46–0.79)	C4, mg/mL	0.35 (0.17–0.45)
eGFR, mL/min/1.73m^2^	34.84	ANA	negative
SUN, mg/dL	21 (9–20)	MPO-ANCA	negative
Total protein, g/dL	5.9 (6.7–8.3)	PR3-ANCA	negative
Albumin, g/dL	2.6 (3.8–5.1)	Anti-GBM antibody	negative
AST, U/L	19 (10–40)	Anti-SS-a/b antibody	negative
ALT, U/L	9 (5–45)	RF	negative
LDH, U/L	229 (120–240)	Cryoglobulin	negative
ALP, U/L	1845 (104–338)	sIL-2R, U/mL	986 (122–496)
γ-GTP, U/L	569 (0–42)	Anti-CL IgG	negative
T-Cho, mg/dL	156 (150–219)	Anti-CLβ2GPI complex	negative
TG, mg/dL	159 (50–150)	ADAMTS13 activity, IU/mL	0.633 (0.780~1.570)
Glucose, mg/dL	124 (75–110)	IL-6, pg/mL	166 (< 8)
HbA1c, %	6.1 (4.6–6.2)	VEGF, pg/mL	494 (< 38.3)
CRP, mg/dL	18.33 (0–0.29)	HBV surface antigen	negative
Urinalysis		HCV antibody	negative
Urine dipstick protein	3+	HIV antibody	negative
Urine occult blood	±	HHV-8 DNA	negative
Spot Urine PCR, g/g	1.57	T-SPOT assay	negative

Abbreviations: *ADAMTS13* A disintegrin-like and metalloproteinase with thrombospondin type 1 motifs 13, *ALP* Alkaline phosphatase, *ALT* Alanine aminotransferase, *ANA* Antinuclear antibody, *ANCA* Antineutrophil cytoplasmic antibody, *APTT* Activated partial thromboplastin time, *AST* Aspartate aminotransferase, *BJP* Bence Jones protein, *CL* Cardiolipin, *Cr* creatinine, *CRP* C-reactive protein, *DNA* Deoxyribonucleic acid, *eGFR* estimated glomerular filtration rate, *FDP* Fibrin degradation products, *GBM* Glomerular basement membrane, *GPI* glycoprotein I, *GTP* Glutamyl transpeptidase, *HbA1c* Hemoglobin A1c, *HBV* Hepatitis B virus, *HCV* Hepatitis C virus, *HHV* Human herpes virus, *HIV* Human immunodeficiency virus, *IgA* Immunoglobulin A, *IgE* Immunoglobulin E, *IgG* Immunoglobulin G, *IgG4* Immunoglobulin G4, *IgM* Immunoglobulin M, *IL* Interleukin, *LDH* Lactate dehydrogenase, *MPO* Myeloperoxidase, *PCR* Protein-creatinine ratio, *PR3* Proteinase 3, *PT* Prothrombin time, *RF* Rheumatoid factor, *sIL-2R* Soluble interleukin-2 receptor, *SS* Sjoegren syndrome, *SUN* Serum urea nitrogen, *T-Cho* Total cholesterol, *TG* Triglyceride, *VEGF* Vascular endothelial growth factor, *WBC* White blood cell

### Kidney biopsy findings

Kidney biopsy was performed at the 6th hospital day before the treatment.

#### Light microscopy

**Fig. 1:**
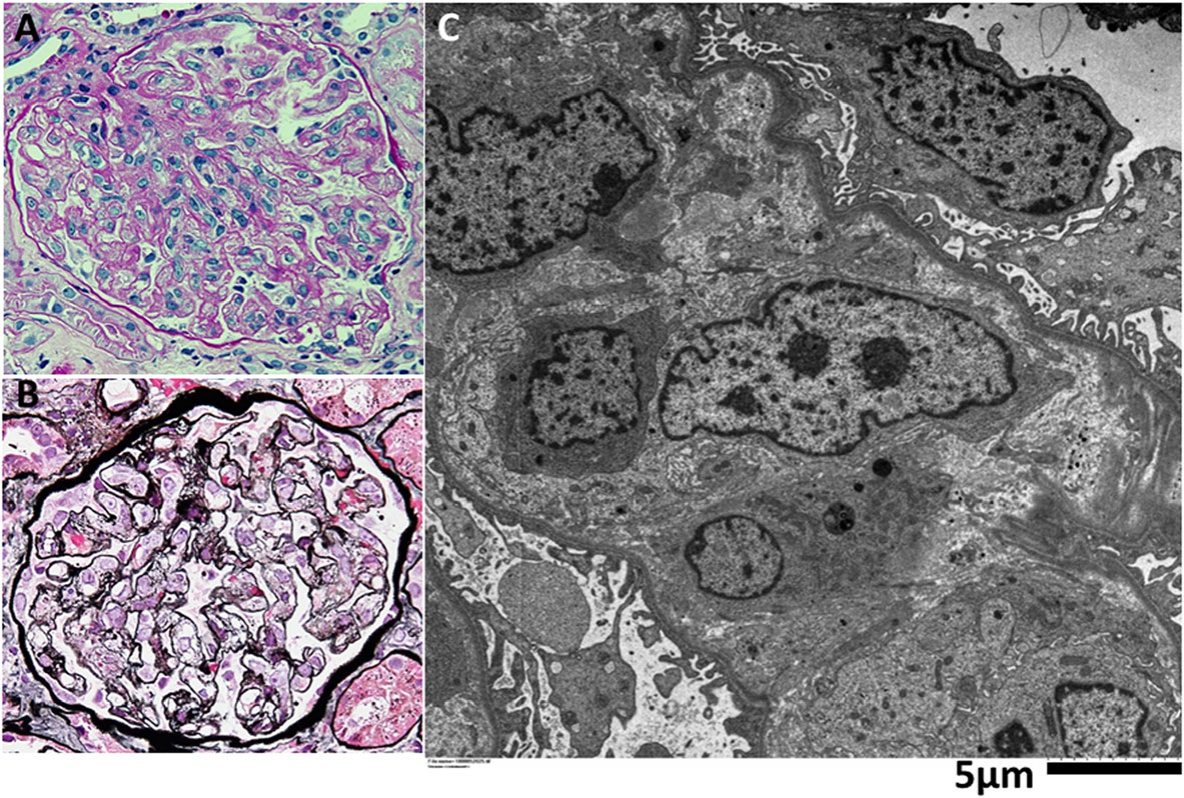
Kidney Biopsy Findings. Periodic-acid-Schiff staining section shows (**a**) diffuse global endocapillary proliferative changes with endothelial swelling in the glomerulus. Periodic acid-silver- methenamine staining section shows (**b**) double contours of partial capillary walls and mesangiolysis. Electron microscopy findings (**c**). There was marked edema in the subendothelial space and in the mesangial area. There were no electron dense deposits. Epithelial cells showed partial foot process effacement and microvillous transformation. (Original magnification, **a**-**b**,× 400)

There were 25 glomeruli present in 2 cores, of which 1 glomerulus was globally sclerosed. In periodic-acid-Schiff staining, glomeruli showed diffuse global endocapillary proliferative changes with endothelial swelling and some infiltration of macrophages (Fig. 1a). Periodic acid-silver- methenamine staining revealed double contours of partial capillary walls and mesangiolysis (Fig. 1b). There was no hyalinosis, segmental sclerosis, or fibrin thrombi. Bowman’s space had no adhesions, fibrin, or crescents. The interstitium showed few focal cell infiltrates, and there was mild tubular atrophy and interstitial fibrosis. Arteries showed mild sclerosis of the intima.

#### Immunofluorescence microscopy

Immunofluorescence was negative for IgG, IgA, IgM, C1q, C3c, C4, κ, λ, and fibrinogen.

#### Electron microscopy (Fig. 1c)

There was marked edema in the subendothelial space and in the mesangial area. There were no electron dense deposits. Epithelial cells showed partial foot process effacement and microvillous transformation.

#### VEGF-A, CD34, and D2–40 staining

**Fig. 2:**
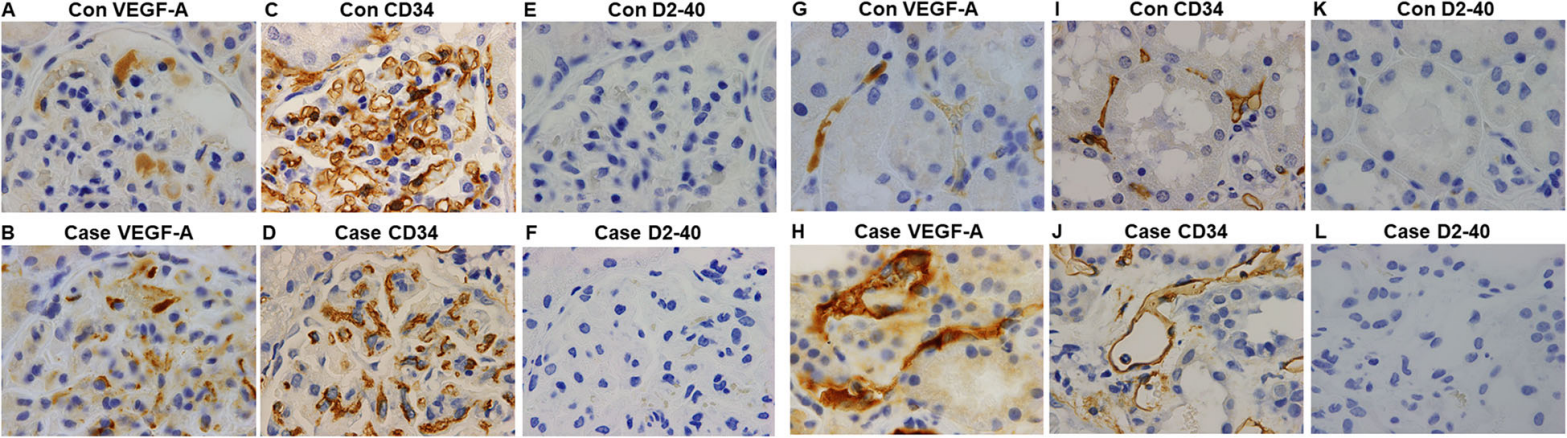
VEGF-A, CD34, and D2–40 staining in kidney biopsy. Glomerular VEGF-A was mainly positive in podocytes both, in the control (**a**) and in the case (**b**) with no significant difference. Cortical VEGF-A positive staining area was significantly increased in our case (**h**) than in the control (**g**). CD34, a marker of endothelium, was positive in glomerular and peritubular capillaries, and arterioles both, in the control (**c** and **i**) and in the case (**d** and **j**). Both glomerular and cortical CD34 positive staining area were significantly decreased in our case compared to the control. D2–40, a marker of lymphatic vessels, was negative in the glomerulus both in the control (**e**) and in the case (**f**). In addition, D2–40 was negative in the targeted cortex area both in the control (**k**) and in the case (**l**). Each Fig. 2a, c, or e shows a same glomerulus in the control and each figure (**b**), (**d**), or (**f**) shows a same glomerulus in our case. Each Fig. 2g, i, or k shows a same cortical interstitium area in the control and each Fig. 2h, j, or l shows a same cortical interstitium area in the case. In the cortical interstitium of the case, VEGF-A was mainly positive in the peritubular capillaries but not in the lymphatic vessels. (Original magnification,× 1000)

An additional file shows the immunodetection and statistical methods (see Additional file 1). We performed immunohistological staining of VEGF-A, CD34, a marker of endothelium, and D2–40, a marker of lymphatic vessels in our case and a normal control kidney (a normal portion of a resected kidney in a patient with renal cell carcinoma) and quantitatively analyzed it (Table 2). Glomerular VFGF-A was especially positive in podocytes both, in the control (Fig. 2a) and in the case (Fig. 2b), with no significant difference in the VEGF-A positive staining area in glomeluri (%) between the control and the case (1.6 ± 0.40 vs. 2.0 ± 0.55, respectively, *P* = 0.63). However, VEGF-A positive staining area in the renal cortex (%) was significantly increased in our case (Fig. 2h) than in the control (Fig. 2g) (1.6 ± 0.31 vs. 0.32 ± 0.072, respectively, *P* <  0.0001). CD34 was positive in glomerular and peritubular capillaries, and arterioles both, in the control (Fig. 2c and i) and in the case (Fig. 2d and j). Glomerular CD34 positive staining area (%) was significantly decreased in our case (Fig. 2d) compared to the control (Fig. 2c) (5.4 ± 0.48 vs. 22 ± 1.2, respectively, *P* <  0.0001). CD34 positive staining area in the renal cortex (%) was also significantly decreased in our case (Fig. 2j) compared to the control (Fig. 2i) (2.5 ± 0.23 vs. 7.1 ± 0.29, respectively, *P* <  0.0001). D2–40 was negative in the glomerulus both in the control (Fig. 2e) and in the case (Fig. 2f), and D2–40 was also negative in the targeted cortex area both in the control (Fig. 2k) and in the case (Fig. 2l). In a small part of renal cortex, D2–40 was positive both in the lymphatic vessels of the control (Additional file 2: Figure S1A) and in the case (Additional file 2: Figure S1B), with no significant difference in the D2–40 positive staining area (%) between the control and the case (0.22 ± 0.091 vs. 0.072 ± 0.030, respectively, *P* = 0.39). Each Fig. 2a, c, or e shows a same glomerulus in the control and each Fig. 2b, d, or f shows a same glomerulus in our case. Each Fig. 2g, i, or k shows a same cortical interstitium area in the control and each Fig. 2h, j, or l shows a same cortical interstitium area in the case. In the cortical interstitium of the case, VEGF-A was mainly positive in the peritubular capillaries but not in the lymphatic vessels.

**Table 2:** Immunohistological analysis in the control and in the case

	Control	Case	*P*-value
VEGF-A positive area in glomeruli (%)	1.6 ± 0.40	2.0 ± 0.55	0.63
VEGF-A positive area in cortex (%)	0.32 ± 0.072	1.6 ± 0.31	< 0.0001
CD34 positive area in glomeruli (%)	22 ± 1.2	5.4 ± 0.48	< 0.0001
CD34 positive area in cortex (%)	7.1 ± 0.29	2.5 ± 0.23	< 0.0001
D2–40 positive area in cortex (%)	0.22 ± 0.091	0.072 ± 0.030	0.39

### Clinical course

**Fig. 3:**
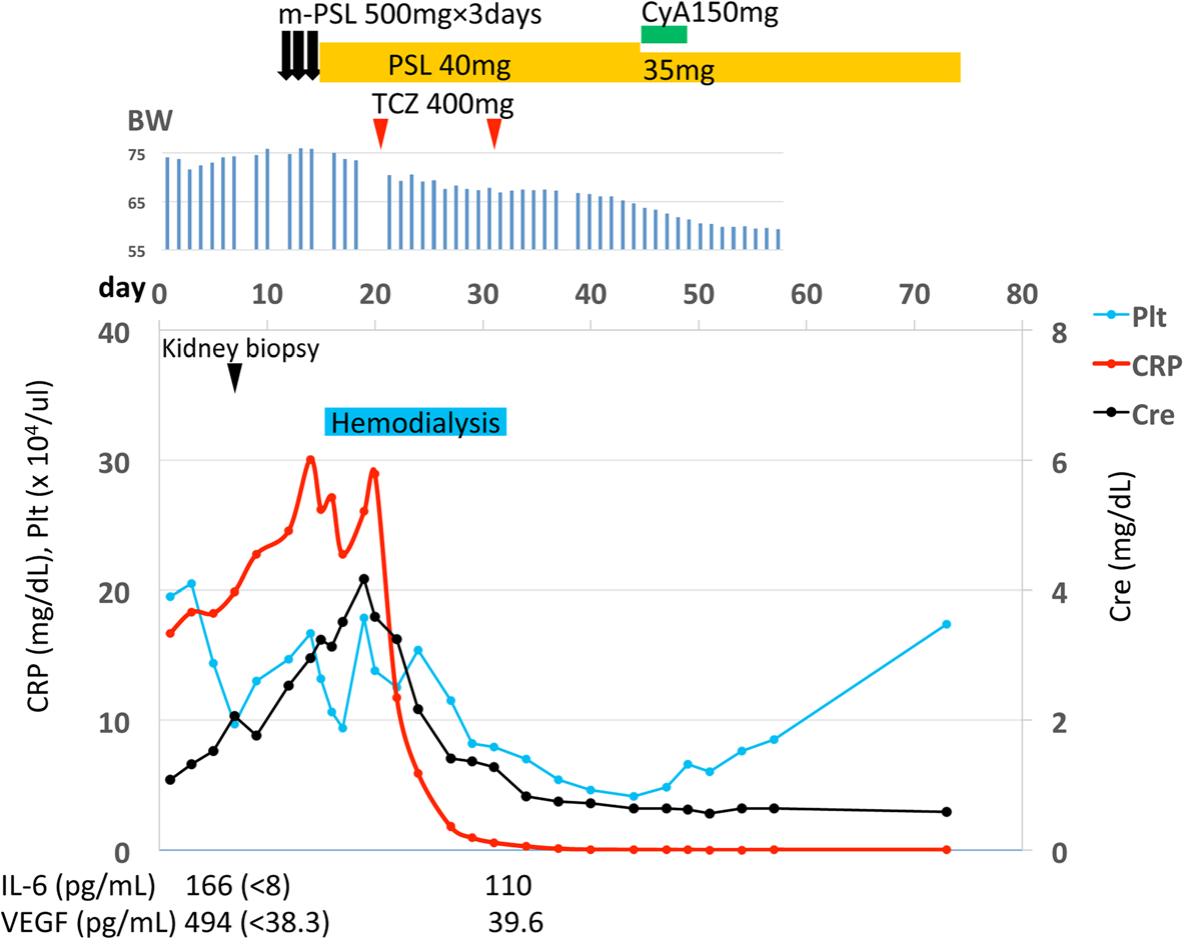
Clinical course of the patient. After the diagnosis of TAFRO syndrome, steroid pulse therapy (500 mg/day of intravenous methylprednisolone) was initiated for 3 days from the 11th hospital day. Thereafter, 40 mg/day of prednisolone was administered orally. However, she developed anasarca, renal dysfunction, and oliguria. Hemodialysis was required from the 15th hospital day. Moreover, serum CRP level remained high, and she experienced considerable painful; therefore, treatment with an anti-IL-6 receptor antibody (tocilizumab) was started at a dose of 8 mg/kg (400 mg/day). Her pain reduced considerably; there was gradual improvement in her condition with respect to renal function and edema. Tocilizumab was administered again after 2 weeks. There was an increase in the urinary volume about 2 weeks after the tocilizumab therapy, and hemodialysis was discontinued. Serum VEGF and IL-6 levels after the second tocilizumab therapy were lower at 39.6 pg/mL (normal < 38.3) and 110 pg/mL (normal < 8), respectively. After the dose of prednisolone was tapered to 35 mg/day, 150 mg/day of cyclosporine was administered orally. However, oral cyclosporine was stopped because of adverse effects such as liver dysfunction and vomiting. She was discharged on hospital day 58. Abbreviations: BW: body weight; Cre: Creatinine; CRP: C-reactive protein; CyA: cyclosporine; IL: Interleukin; m-PSL: methylprednisolone; Plt: platelets; PSL: Prednisolone; TCZ: tocilizumab; VEGF: Vascular endothelial growth factor

The clinical course is illustrated in Fig. 3. Anasarca, including pleural effusion, ascites, and general edema gradually deteriorated. Biopsy of the porta hepatis lymph node with endoscopic ultrasound-fine needle aspiration (EUS-FNA) was performed at the 11th hospital day before the treatment. There was no evidence of Castleman disease or malignant lymphoma. Just before treatment initiation, her platelet count was < 100 × 10^3^/μL. Based on the criteria [2], her clinical and laboratory findings fulfilled the diagnostic criteria for TAFRO syndrome. A bone marrow biopsy revealed a normocellular marrow with increased number of megakaryocytes, without myelofibrosis. Steroid pulse therapy (500 mg/day of intravenous methylprednisolone) was initiated for 3 days from the 11th hospital day. Thereafter, 40 mg/day of prednisolone was administered orally. However, she developed anasarca, renal dysfunction, and oliguria. Hemodialysis was required from the 15th hospital day. Moreover, serum CRP level remained high, and she experienced considerable painful; therefore, treatment with an anti-IL-6 receptor antibody (tocilizumab) was started at a dose of 8 mg/kg (400 mg/day). Her pain reduced considerably; there was gradual improvement in her condition with respect to renal function and edema. Tocilizumab was administered again after 2 weeks. There was an increase in the urinary volume about 2 weeks after the tocilizumab therapy, and hemodialysis was discontinued. Serum VEGF and IL-6 levels after the second tocilizumab therapy were lower at 39.6 pg/mL and 110 pg/mL, respectively. After the dose of prednisolone was tapered to 35 mg/day, 150 mg/day of cyclosporine was administered orally. However, oral cyclosporine was stopped because of adverse effects such as liver dysfunction and vomiting. She was discharged on hospital day 58. About 1 year after discharge, prednisolone had been tapered to 5 mg/day, and her renal outcome was stable (final serum creatinine level was 0.70 mg/dL and urinary protein was negative).

## Discussion and conclusions

Several studies have examined renal biopsy findings in TAFRO syndrome (Table 3) [4–13]. Renal histology in the 10 patients [4–13] and our patient mainly showed MPGN-like lesions or TMA-like glomerulopathy. The timing of the kidney biopsy performed in these patients varied from the acute phase [8–13] to the chronic phase [4–7]; however, glomerular microangiopathy was a common finding. We explored the details of kidney biopsy findings in the literature review and found that 3 (Case no.3 and 5–6, Table 3) out of 5 cases diagnosed as MPGN-like glomerulopathy had no glomerular immune deposits. We could diagnose these three cases as TMA-like glomerulopathy. If so, all patients with kidney biopsy performed in the acute phase (Case no. 5–10, Table 3 and our case) could be diagnosed as TMA-like glomerulopathy. On the other hand, in four patients with kidney biopsy performed in the chronic phase (Case no. 1–4, Table 3), two patients (Case no.1 and 2, Table 3) were diagnosed as MPGN-like glomerulopathy and the others (Case no.3 and 4, Table 3) could be diagnosed as TMA-like glomerulopathy. In TAFRO syndrome, we suspect that TMA appears in acute phase and the renal histology shows MPGN in chronic phase. In the 11 cases of TAFRO syndrome with kidney biopsy, there was no clear evidence of systemic microangiopathy including hemolytic anemia or organ dysfunction except kidney. Local glomerular microangiopathy was characteristic of these patients with TAFRO syndrome.

**Table 3:** Clinical characteristics of 11 cases of TAFRO syndrome with kidney biopsy

Case no./Ref.	Age/sex	UP	Alb (g/dL)	Cr (mg/dL)	CRP (mg/dL)	VEGF/IL-6 (pg/mL)	1st Therapy/ response	2nd Therapy/ response	Kidney biopsy findings									
									Thrombi	Endothelial swelling	Endocapillary proliferation	Mesangiolysis	Mesangial proliferation	Double contours of GBM	Glomerular Ig deposition by IF	Subendothelial lesions by EM	Electron dense deposits	Diagnosis by authors
1/ [4]	38 / M	n.d.	3	2.59	11.18	4420 / n.d.	PSL / good	-	n.d.	n.d.	n.d.	n.d.	Yes	Yes	Yes	n.d.	n.d.	MPGN-like
2/ [5]	55 / F	0.54 (g/g・Cr)	2.7	2.1	4.1	464 / 11.7	Pulse steroid / good	-	n.d.	n.d.	n.d.	n.d.	Yes	Yes	Yes	n.d.	No	MPGN-like
3/ [6]	76 / F	0.30 (g/day)	1.2	3.02	16	1350 / 49.2	PSL /good	-	n.d.	n.d.	Yes	n.d.	Yes	Yes	No	Yes	No	MPGN-like
4/ [7]	79 / F	2.65 (g/g・Cr)	2.4	1.85	3.9	15.6 / 3.76	Pulse steroid, PE / bad	RTX / good	n.d.	n.d.	No	Yes	No	Yes	No	Yes	n.d.	TMA-like
5/ [8]	70 / M	0.33 (g/day)	2.5	1.28	9.85	126 / 33	Pulse steroid / good	-	No	Yes	No	Yes	No	Yes	No	Yes	No	MPGN-like
6/ [9]	61 / F	n.d.	2.8	2.14	23.12	n.d. / 722.6	Pulse steroid, TCZ, RTX / good	-	n.d.	Yes	n.d.	Yes	Yes	Yes	No	n.d.	n.d.	MPGN-like
7/ [10]	80 / F	0.41 (g/day)	2.8	1.17	7.3	454 / 21.6	PSL /bad	TCZ / good	No	Yes	Yes	No	No	Yes	Yes	Yes	No	TMA-like
8/ [11]	51 / F	0.52 (g/g・Cr)	2.5	1.03	4.58	198 / 21.2	Pulse steroid / good	-	n.d.	Yes	No	Yes	No	Yes	No	n.d.	n.d.	TMA-like
9/ [12]	84 / M	0.30 (g/day)	2.4	2.31	8.3	177 / 12.3	PSL, PE /bad	TCZ / good	No	Yes	Yes	Yes	No	No	No	Yes	No	TMA-like
10/ [13]	54 / F	3.2 (g/g・Cr)	2.4	1.11	7	n.d. / 8.2	PSL /good	-	No	Yes	No	No	No	No	No	Yes	No	Endothelial injury
our case	48 / F	1.57 (g/g・Cr)	2.6	1.32	18.33	494 / 166	Pulse steroid /bad	TCZ / good	No	Yes	Yes	Yes	No	Yes	No	Yes	No	TMA-like

Abbreviations: *Alb* Albumin; *Cr* Creatinine, *CRP* C-reactive protein, *EM* Electron microscopy, *GBM* Glomerular basement membrane, *IF* Immunofluorescence, *Ig* Immunoglobulin, *IL* Interleukin, *MPGN* Membranoproliferative glomerulonephritis, *n.d.* not data, *PE* Plasma exchange, *PSL* Prednisolone, *RTX* rituximab, *TCZ* tocilizumab, *TMA* Thrombotic microangiopathy, *UP* Urinary protein, *VEGF* Vascular endothelial growth factor

Serum VEGF and IL-6 levels are usually elevated in TAFRO syndrome and are considered pathogenic factors. In our case, serum VEGF and IL-6 levels were decreased, as patient condition improved. In Table 3, all cases except Case no. 4 presented with elevated serum IL-6 and/or VEGF. Therefore, IL-6-induced VEGF overproduction may lead to glomerular endothelial injury [15]. Striking glomerular endothelial injury can rapidly reduce glomerular filtration rates, causing oliguric kidney injury. IL-6-VEGF-axis-induced glomerular microangiopathy may play a crucial role in developing acute kidney injury in TAFRO syndrome. In contrary, VEGF may protect renal TMA. Systemic administration of VEGF accelerated the renal recovery in a rat TMA model [16, 17]. However, systemic inhibition of VEGF signaling with anti-VEGF therapy in six patients caused TMA-like glomerulopathy with endothelial injury, and a mouse model of podocyte-specific VEGF deletion developed TMA-like glomerular lesions [18]. Whether VEGF plays a causative or protective role or both in renal TMA remains unclear and further studies are needed. Moreover, the site of VEGF (systemic or local) that plays an important role in glomerular microangiopathy remains unclear. A podocyte-specific VEGF transgenic mouse showed proteinuria and focal effacement of the podocytes without defects in glomerular endothelial cells on transmission electron microscopy [19]. VEGF-VEGF receptor paracrine signaling between podocytes and glomerular endothelial cells is considered essential for the maintenance of glomerular endothelial cells [19]. Decrease in the glomerular VEGF levels may cause failure of homeostasis in the endothelium and lead to the development of TMA. Either too much or too little VEGF in the glomerulus may lead to glomerular pathology.

TMA-like glomerulopathy was the most common feature of renal involvements in Castleman disease [20]. There were no significant differences in the glomerular VEGF expression among patients with TMA with or without Castleman disease and healthy controls [20]. In some patients with a type of Castleman disease-associated TMA (small-vessel lesion group), glomerular VEGF expression was decreased [20]. However, limited information is available about glomerular VEGF expression in TAFRO syndrome. Glomerular VEGF staining in TAFRO syndrome has been reported only in two cases [10, 11]. One was VEGF negative and the other was VEGF positive. We performed immunohistological staining of VEGF-A, CD34, and D2–40, in our case and a normal control kidney, and quantitatively analyzed it. Glomerular VFGF-A was especially positive in podocytes both, in the control and in the case, with no significant difference, although significant difference of glomerular VEGF-A expression between the control and the case was expected. It is noteworthy that the VEGF-A positive staining area in the renal cortex was significantly increased in our case than in the control. Both glomerular and renal cortical CD34 positive staining area were significantly decreased in our case compared to the control. It is reported that glomerular capillary injuries in acute and chronic glomerular lesions in patients with IgA nephropathy were associated with the loss of CD34 positive glomerular endothelial cells [21], however, the association between TMA and CD34 staining remains unknown. The weakness of CD 34 staining in our case may reflect microangiopathy. In a small part of renal cortex, D2–40 was positive both in the control and in the case with no significant difference. As a result of additional CD34 and D2–40 staining, VEGF-A was mainly positive in the peritubular capillaries, but not in the lymph ducts in the cortical interstitium. VEGF homeostasis that the exact balance of VEGF in the glomerulus and perhaps in the peritubular capillary system as well may be critical. Unfortunately, we were not able to evaluate any differences in peritubular capillary ultrastructure that could help explain pathophysiology. Further studies about renal VEGF expression in TAFRO syndrome are warranted.

By the Japanese diagnostic criteria and treatment strategy of TAFRO syndrome (2015 version) [2], first line therapy for TAFRO syndrome is high-dose glucocorticoid. For TAFRO syndrome refractory to or dependent on glucocorticoids, immunosuppressants including cyclosporine, tocilizumab, and rituximab are considered. In our case, the glucocorticoid therapy, including steroid pulse was ineffective; however, the anti-IL-6 receptor antibody (tocilizumab) therapy was very effective. It is suggested that IL-6-VEGF axis contributed to systemic inflammation and increased vascular permeability in our case. Table 3 shows that glucocorticoid therapy was effective in 6 out of 11 patients with TAFRO syndrome with biopsy-proven MPGN-like or TMA-like glomerulopathy and tocilizumab therapy was effective in 3 out of 4 of the patients resistant to steroids. Tocilizumab therapy may be a useful choice in patients with TAFRO syndrome with MPGN-like or TMA-like glomerulopathy.

In conclusion, we reported a case of TAFRO syndrome with TMA-like glomerulopathy with successful treatment by tocilizumab. We reviewed our case and other 10 previous reports about renal biopsy findings in TAFRO syndrome and found that glomerular microangiopathy was a common finding. IL-6-VEGF-axis-induced glomerular microangiopathy may play a crucial role in developing acute kidney injury in TAFRO syndrome. The anti-IL-6 receptor antibody therapy may be useful for TAFRO syndrome refractory to glucocorticoids. Glomerular VFGF-A was especially positive in podocytes both, in the control and in the case, with no significant difference. However, the VEGF-A positive staining area in the cortical peritubular capillaries was significantly increased in our case than in the control. VEGF homeostasis that the exact balance of VEGF in the glomerulus and perhaps in the peritubular capillary system as well may be critical. Further investigation about the pathophysiology of VEGF in TAFRO syndrome is needed.

## Supplementary information

**Additional file 1.** Immunodetection and Statistical Methods.

**Additional file 2: Figure S1.** In a small part of renal cortex, D2–40 was positive both in the lymphatic vessels of the control (A) and in the case (B).

## Data Availability

Further clinical data and images of this case are available from the corresponding author upon reasonable request.

## Abbreviations

ADAMTS13: A disintegrin and metalloprotease with thrombospondin type 1 motifs 13
CRP: C-reactive protein
eGFR: estimated glomerular filtration rate
EUS-FNA: Endoscopic ultrasound-fine needle aspiration
HHV: Human herpes virus
HIV: Human immunodeficiency virus
IgG4: Immunoglobulin G4
IL: Interleukin
MPGN: Membranoproliferative glomerulonephritis
TMA: Thrombotic microangiopathy
TTP: Thrombotic thrombocytopenic purpura
VEGF: Vascular endothelial growth factor
