# Supplementary material for: TAFRO syndrome as a cause of glomerular microangiopathy: a case report and literature review
Source: BMC Nephrol. 2019 Oct 17;20:375. doi: 10.1186/s12882-019-1574-9 (PMC6798393; doi:10.1186/s12882-019-1574-9)
Supplement: Supplementary file 1 — Additional file 1. Immunodetection and Statistical Methods. [file 12882_2019_1574_MOESM1_ESM.docx]

**Immunodetection and Statistical Methods.**

Primary antibodies used are as follows: a monoclonal mouse antibody to VEGF-A (clone VG-1, Abcam, Cambridge, England), a monoclonal mouse antibody to CD34 (clone NU-4A1, Nichirei, Tokyo, Japan), and a monoclonal mouse antibody to D2-40 (clone D2-40, Nichirei, Tokyo, Japan). Kidney tissues were ﬁxed over 24–30 h at room temperature in 10% buffered formalin. Two-micrometer formalin ﬁxed parafﬁn sections were treated by an antigen retrieval system using HEAT PROCESSOR Solution pH9 (Nichirei, Tokyo, Japan) and HEAT PROⅡ(Nichirei, Tokyo, Japan). The paraffin sections were stained by an automated staining system using HISTSTAINER (Nichirei, Tokyo, Japan). Primary antibodies were incubated for 30 minutes at room temperature. Detection of bound primary antibody was achieved by using HISTOFINE SIMPLE STAIN MAX-PO (MULTI) (Nichirei, Tokyo, Japan) with 3,3-diaminobenzidine (DAB) as substrate.

VEGF-A, CD34, and D2-40 expression were quantiﬁed using Image J software version 1.45, and the positive staining area was calculated as the percentage of total area as previously described ^1^. Fourteen glomeruli and twelve cortex areas were evaluated both, in the control and in the case. For statistical analysis, values are means±SEM. Statistical signiﬁcance was evaluated using GraphPad Prism, version 7.0 (GraphPad Software, San Diego, CA). Mann-Whitney test was applied to all analyses.

Reference

1. Nagayama Y, Braun GS, Jakobs CM, e al. Gp130-dependent signaling in the podocyte.

Am J Physiol Renal Physiol. 2014;307(3): F346-F355.
