# Supplementary material for: TAFRO syndrome as a cause of glomerular microangiopathy: a case report and literature review
Source: BMC Nephrol. 2019 Oct 17;20:375. doi: 10.1186/s12882-019-1574-9 (PMC6798393; doi:10.1186/s12882-019-1574-9)

**Figure S1.** D2-40 positive staining in the renal cortex

D2-40 was positive in the lymphatic vessels both in the control (A) and in the case (B) with no significant difference. (Original magnification,×400 )


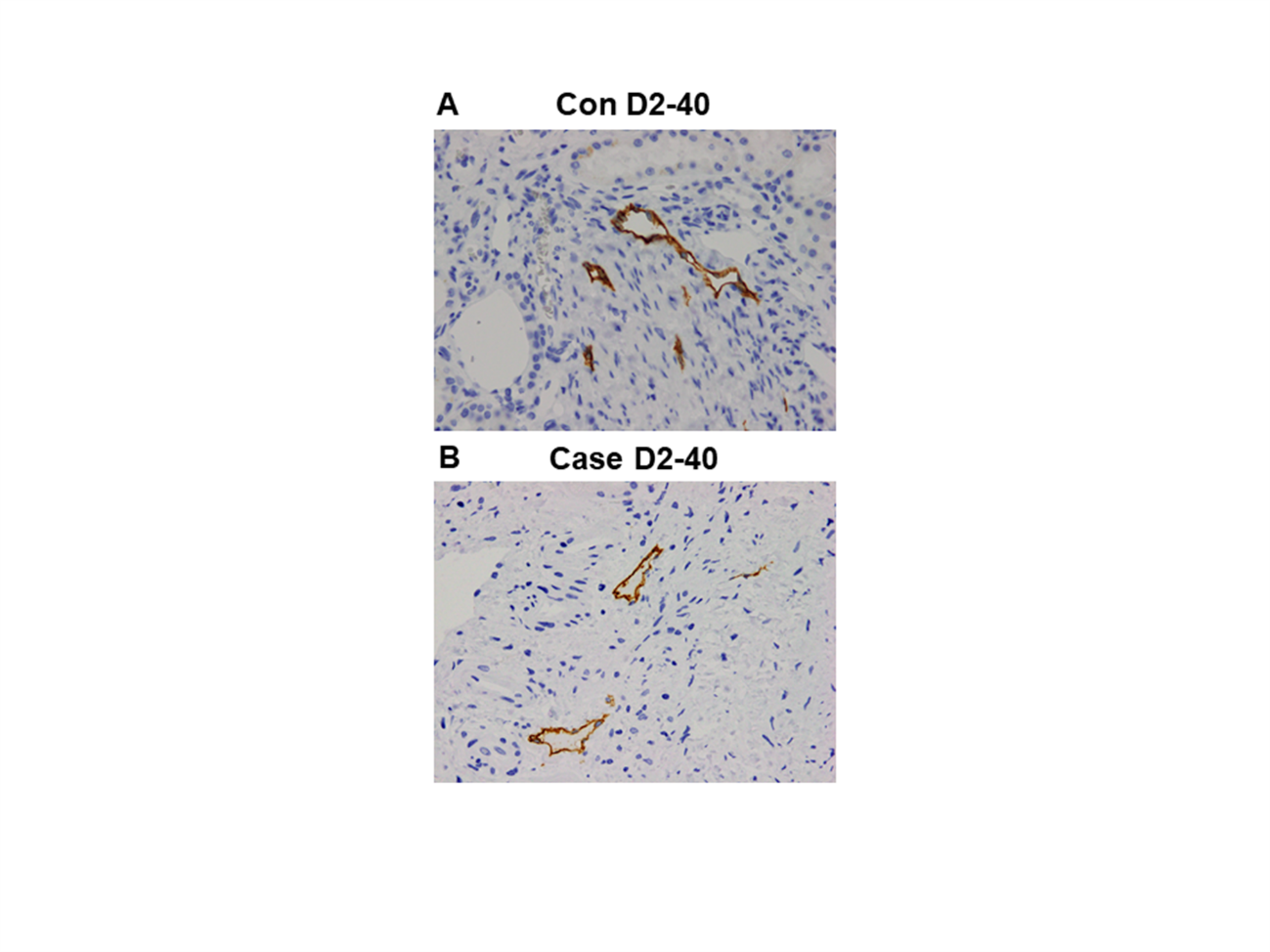

Supplement: Supplementary file 2 — Additional file 2: Figure S1. In a small part of renal cortex, D2–40 was positive both in the lymphatic vessels of the control (A) and in the case (B). [file 12882_2019_1574_MOESM2_ESM.docx]
